# Auger effect in weakly confined nanocrystals

**DOI:** 10.1038/s41377-023-01227-x

**Published:** 2023-07-24

**Authors:** Jiawen Liu, Laurent Coolen

**Affiliations:** 1 Laboratoire de Physique de l’Ecole Normale Supérieure, ENS, Université PSL, CNRS, Sorbonne Université, Université Paris Cité, F-75005 Paris, France; 2 Sorbonne Université, CNRS, Institut de NanoSciences de Paris, INSP, F-75005 Paris, France

**Keywords:** Optical physics, Optical materials and structures

## Abstract

An extensive analysis of biexciton luminescence in high-quality, large perovskite CsPbBr_3_ nanocrystals shows how the biexciton Auger decay rate deviates from the “universal” volume scaling as the exciton confinement becomes weaker.

## Abstract (web summary)

As the confinement of excitons weakens, an investigation of biexciton luminescence in large perovskite nanocrystals reveals a deviation in the biexciton Auger decay rate from the universal volume scaling.

## Graphical abstract

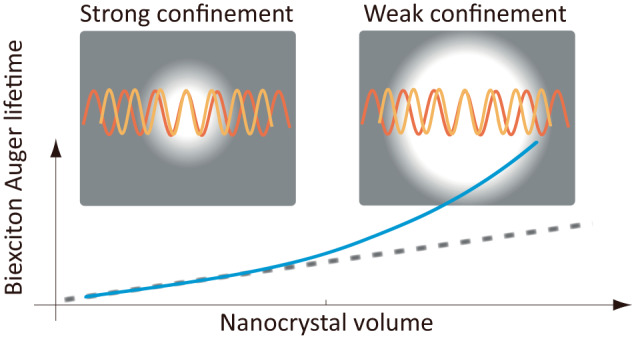

The Auger effect in semiconductor nanoparticles causes nonradiative recombination of electron-hole pairs and significantly impacts their photophysical behavior. Improving the fundamental understanding of the Auger effect is imperative and remains an active field of research. Previous works have primarily focused on nanocrystals in the strong confinement regime, while the weak confinement regime in intermediate-sized systems is less explored due to several long-standing challenges. In a recent report^1^, Peng Huang and co-authors describe high-quality perovskite nanocrystals in the weak confinement regime. They experimentally reveal a superlinear increase in Auger recombination time with nanocrystal volume and provide a successful interpretation using a nonlocal interaction model. This work challenges the universal volume scaling law^2,3^ and deepens our fundamental understanding of the Auger effect at the limit between bulk and confined materials.

Semiconductor nanocrystals, also known as quantum dots, have attracted significant attention in recent decades. Apart from the high degree of control over their composition and shape, the size of nanocrystals can be manipulated at the atomic level, giving rise to the quantum confinement of electrons and holes. This effect results in discrete energy levels and size-tunable bandgap, allowing for the absorption and emission of light across a wide range of wavelengths. Nanocrystals are thus highly attractive for applications in optoelectronic devices such as light-emitting diodes (LEDs), photodetectors and photovoltaic solar cells.

Upon light absorption in a semiconductor, an electron and a hole in a bound state, known as an exciton, are generated in nanocrystals. If a third charge (electron or hole) is present, excitonic radiative recombination can be bypassed through Auger recombination, a nonradiative coulomb interaction by which an exciton recombines and yields its energy to a spectator charge. Consequently, the behavior of excitons is strongly influenced by the Auger effect, particularly in nanocrystals, due to the spatial confinement of charge carriers. For optoelectronic applications, the Auger effect is mostly detrimental because it causes nonradiative recombination processes that compete with luminescent processes, reducing the quantum yield of photoluminescence or generating intermittent fluctuations (blinking) in emission intensity. The Auger effect also impacts the charge carrier dynamics, leading to carrier relaxation, trapping, recombination, and energy transfer that compete with the desired charge extraction process. On the other hand, there are cases where the Auger effect can be favorable. A prominent example is in single-photon sources for quantum information, where the Auger effect helps suppress multi-photon emission processes by efficiently transferring the excess energy to another carrier so that photons are always emitted one by one in smaller nanoparticles.

Therefore, understanding and controlling the Auger effect in nanocrystals is pivotal for the performance of nanocrystal-based devices. One topic of debate in the literature is the size-dependence of the Auger decay time: most often, a “universal” volume scaling law is found, but several authors reported deviations from this law^4,5^. In addition, previous studies mostly focused on the strong confinement regime where the dimension of the nanocrystal is smaller than the exciton Bohr radius ${a}_{B}$ (this is the regime where the coulombic electron-hole binding energy is much lower than confinement effects so that the electron and hole are described with better relevance as two separate particles rather than as an exciton). However, there is a significant lack of exploration regarding the Auger effect in the weak confinement regime. This is primarily due to the difficulties in fabricating high-quality large nanoparticles for unambiguous analysis, where the Auger recombination is the dominant nonradiative decay without substantial perturbation induced by defects or other factors. Moreover, accurately modeling these recombination processes is challenging because of the intricate interplay between carrier energies, wave functions, and interaction strengths.

In the article by Huang and co-authors^1^, a comprehensive approach has been undertaken combining advanced materials synthesis strategies, experimental analysis at both ensemble and single-emitter scales, and modeling considering the nonlocal effect. Inorganic perovskite CsPbBr_3_ nanocrystals were considered, in which the limit between confined and non-confined structures, as given by the exciton wavefunction period λ_X_ (de Broglie wavelength), is estimated as 14 nm (or 370 nm^3^ in volume), while the Bohr radius is 7 nm^6^. High-quality CsPbBr3 samples in spheroidal or rod shapes were prepared with various volumes from 1000 nm^3^ up to 10000 nm^3^ (thus well positioned within the weak confinement domain). The photoluminescence decay dynamics, excitation power dependence, blinking trajectories and photon correlations were systematically investigated so that the radiative contribution could be isolated from the total biexciton decay rate and the Auger decay rate was extracted. The data analysis demonstrates that the Auger recombination lifetime is exponentially increased in large nanocrystals instead of obeying the “universal” volume scaling law. The origin of this exponential “nonlocal” term is quantitatively well explained by a concise model, where the Auger decay of the exciton (treated as a point particle: weak confinement regime) scales as the overlap between the wave functions of the initial and final states of the spectator hole charge. The nonlocal effect appears when the wave vector difference ${k}_{i}-\,{k}_{f}=\,2\pi /{\lambda }_{X}$ affects this overlap integral, which occurs when the nanocrystal radius *R* is larger than ${\lambda }_{X}$ (Fig. 1).

**Fig. 1:**
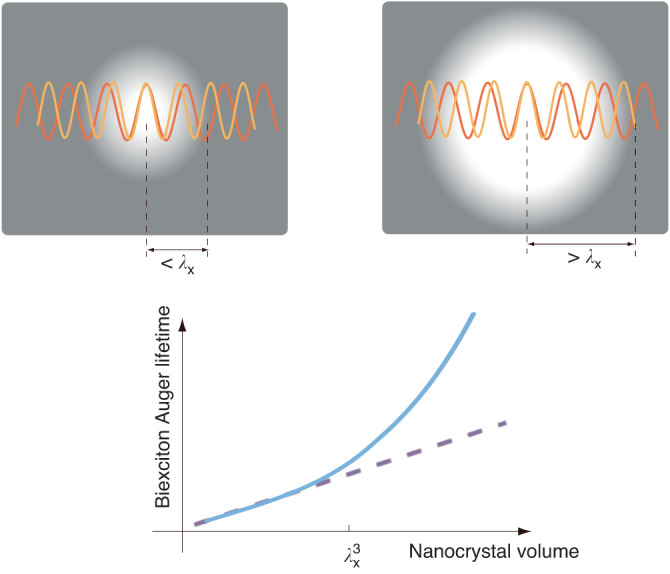
Illustration for the appearance of nonlocal effects. As the nanocrystal size increases above the exciton wavefunction period λ_X_, the biexciton Auger decay becomes much slower than predicted by the volume scaling law because the phase difference between the initial and final states of the spectator hole leads to an inhibition of the Auger mechanism

Due to the suppressed Auger effect, exceptionally high biexciton efficiency reaching 80% is demonstrated in the present work. While this result may not directly promote the development of entangled photon sources, it is indeed significant for the fundamental understanding of the Auger effect. High-quality perovskite nanocrystals thus appear as a promising platform for the analysis and tuning of Auger energy transfers. Whereas the present paper compares large nanocrystals with literature data on smaller particles, future improved control on perovskite nanocrystal synthesis should provide direct comparisons between the strongly, weakly and non-confined regimes. Then a more general model will be needed to account for both $R/{a}_{B}$ and $R/{\lambda }_{X}$ ratios. The nanoparticle geometry provides further degrees of freedom to achieve specific confinement regimes and test theoretical predictions, as exemplified recently in CdSe nanoplatelets^7^.
